# Editorial: Molecular Mechanisms of Multiple Myeloma

**DOI:** 10.3389/fonc.2022.870123

**Published:** 2022-03-10

**Authors:** Alessandro Gozzetti, Chung Hoow Kok, Chien-Feng Li

**Affiliations:** ^1^ Hematology Unit, University of Siena, Azienda Ospedaliero Universitaria Senese, Siena, Italy; ^2^ Adelaide Medical School, Faculty of Health and Medical Sciences, University of Adelaide, Adelaide, SA, Australia; ^3^ National Institute of Cancer Research, National Health Research Institutes, Miaoli, Taiwan

**Keywords:** multiple myeloma, genetics of myeloma, extramedullary, biology, signaling

Multiple myeloma (MM) is a plasma cell disorder representing the second most common blood cancer (1). MM is still defined as an incurable disease, but survival has nearly doubled in latest years due to novel drugs and novel therapeutic strategies (Gozzetti et al., 2–4). Also, high-risk MM had benefited from novel therapies, although with less potency (5–8). Knowledge of the molecular mechanisms and pathogenesis of MM is behind this progress, in particular genetics of the monoclonal plasma cells and their interactions with the microenvironment (9, 10).

MM cell proliferation and apoptosis are touched by the paper entitled “Study of Tim3 regulation in multiple myeloma cell proliferation *via* NF-kB signal pathways” (Liu et al.). T-cell immunoglobulin and mucin domain-3 (Tim3) is a negative regulatory factor of cellular immunity (11). In this study, Liu et al. found a higher expression of Tim3 by flow cytometry in bone marrow plasma cells derived from 167 MM patients when compared with 51 healthy donors. Additionally, higher Tim3 expression level was associated with poorer prognostic factor based on International Staging System (ISS) stage III when compared to stage I and II. Mechanistically, the authors showed that cell proliferation was decreased, and apoptosis was induced *via* NF-kB signaling upon Tim3 knocked-down *in vitro* by siRNA using two MM cell lines. Furthermore, the authors observed that Tim3 knockdown used in combination with the anti-myeloma therapy bortezomib had an additive effect on apoptosis in MM cell lines. This suggests that Tim3 may be a potential therapeutic target.

Host immunity is crucial in antitumor activity. In the paper entitled “Metabolic reprogramming induces immune cell dysfunction in the tumor microenvironment of multiple myeloma”, Wu et al. review data about metabolic reprogramming in MM, which is associated with the hypoxic, acidic, and nutritionally deficient microenvironment. In particular, authors remark how these findings can negatively impact the anti-tumor activity of the immune cells, i.e. T-cell mediated tumor lysis *via* silencing of PTPN1, TP53I11 induced by hypoxia (12, Wegiel et al.), reduced NK activity by decreased ligand receptors RAE-1 and PVR on MM cells (13) and PD-L1 upregulation *via* HIF-1a (14).

Metabolic abnormalities are important in cancer, age, obesity can be cancer-promoting factors and can affect also disease responsiveness and progression. Lazaris et al. in the paper “The lipoprotein transport system in the pathogenesis of multiple myeloma: advances and challenges,” review the role of bone marrow adipocytes to support growth and proliferation of MM plasma cells and bone remodeling (15, 16). The deregulation of the lipoprotein system seems to correlate with MM development together with obesity. Interestingly, different studies looked at serum lipid assessment in MM patients during treatment and one found higher APOA1 (the major apolipoprotein of high-density lipoprotein HDL) related to better survival (17–19).

Methylation has been reported to be present in MM (20), although hypomethylating agents are not very much used in clinical practice. The paper “KDM2A targets PFKB3 for ubiquitylation to inhibit the proliferation and angiogenesis of multiple myeloma cells” by Liu et al. showed that the lysine demethylase KDM2A acts not only as an epigenetic regulator in cancer but also as an inhibitor of MM plasma cells proliferation and angiogenesis through ubiquitination of PFKB3, a crucial enzyme in glycolysis (21, 22). Moreover, IL-32 and the vascular endothelial growth factor (VEGF), direct key players in promoting angiogenesis, were measured and found increased in knockdown KDM2A MM cells. These findings suggest KDM2A ubiquitination of PFKB3 as a possible therapeutic target in myeloma.

Extramedullary MM (EMM) represents an unmet clinical need in daily practice (23–25). Even though new drugs increased the percentage of responses in this field, the prognosis is still poor. Much remains unknown on the molecular basis of EMM. In the last article, “Intratumor heterogeneity of MIF expression correlates with extramedullary involvement in myeloma,” Xu et al. highlight the role of MIF (macrophage migration inhibitory factor) expression in the development of EMM. In particular, authors found low levels of MIF expression in extramedullary biopsies of 17 patients compared to intramedullary biopsies. MIF high expression induced high proliferation of MM cells in *in vivo* mouse models, suggesting a role for MIF in EMM.

Altogether these studies highlight the different molecular mechanisms of MM development and aggressiveness and suggest a possible new target for MM therapy.

## Author Contributions

All authors listed have made a substantial, direct, and intellectual contribution to the work and approved it for publication.

## Conflict of Interest

The authors declare that the research was conducted in the absence of any commercial or financial relationships that could be construed as a potential conflict of interest.

## Publisher’s Note

All claims expressed in this article are solely those of the authors and do not necessarily represent those of their affiliated organizations, or those of the publisher, the editors and the reviewers. Any product that may be evaluated in this article, or claim that may be made by its manufacturer, is not guaranteed or endorsed by the publisher.
